# Examining an Evolving Biologically Inspired Design Professional Learning Environment through Conjecture Mapping and Design-Based Research

**DOI:** 10.3390/biomimetics9080468

**Published:** 2024-08-02

**Authors:** Abeera P. Rehmat, Alexandra A. Towner, Meltem Alemdar, Michael E. Helms, Jeffrey H. Rosen, Roxanne A. Moore, Marc J. Weissburg

**Affiliations:** 1 Center for Education Integrating Science, Mathematics, and Computing, Georgia Institute of Technology, Atlanta, GA 30332, USAmeltem.alemdar@ceismc.gatech.edu (M.A.); jrosen3@mail.gatech.edu (J.H.R.); roxanne.moore@gatech.edu (R.A.M.); 2 George W. Woodruff School of Mechanical Engineering, Georgia Institute of Technology, Atlanta, GA 30332, USA; michael.helms@me.gatech.edu; 3 School of Biological Sciences, Georgia Institute of Technology, Atlanta, GA 30332, USA; marc.weissburg@biology.gatech.edu

**Keywords:** teacher professional learning, biologically inspired design, conjecture mapping, design-based research, pre-college engineering

## Abstract

Biologically inspired design (BID) in engineering is a convergent, systematic approach that uses analogies from biological organisms to develop solutions for human engineering and design problems. Based on outcomes from prior studies of integrating BID in higher education, incorporating BID into pre-college education is a logical evolution. For effective BID instruction of these convergent concepts in pre-college education, teachers need to be well-equipped with biological, engineering, and pedagogical knowledge, both in general and those unique to the convergent, still evolving discipline. In this paper, we investigate the Professional Learning (professional learning) environment designed to foster engineering teachers’ understanding of BID integration in engineering and to determine to what extent the evolving professional learning environment fostered engineering teachers’ conceptual knowledge of BID across the three-year project. This design study applies conjecture mapping with design-based research (DBR) to examine a professional learning environment that changed over three summers and its impact on teachers’ conceptual understanding of BID integration in engineering. The analysis indicates that a combination of experiential and informal learning experiences along with engagement in a formal design challenge promoted teacher enthusiasm and a conceptual understanding of BID across the three years. Professional learning fostered teachers’ understanding of BID integration in engineering and enabled them to integrate BID into their engineering teaching practice.

## 1. Introduction

Biologically inspired design (BID) is a design-by-analogy technique that uses biological analogies to inspire unique solutions to complex design problems [1]. Biologically inspired design has emerged as an area of interest in education due to its ability to inspire innovation and new potential design solutions, as demonstrated by a dramatic growth in BID-related patents [2,3]. When applied to human problems, the results are often economical, elegant, and sustainable [4]. BID is a powerful and logical bridge to multidisciplinary and convergence education [4], increasing in prevalence within graduate and undergraduate engineering education. The integration of BID is also consistent with a heightened emphasis within engineering to develop cross-disciplinary skills and adaptive, sustainable design [5,6]. In addition, the incorporation of BID into engineering education may also have secondary benefits to engineering. For example, BID inclusion is believed to be more appealing to students typically underrepresented in the engineering field, particularly women, as they tend to show greater interest in interdisciplinary content [6,7,8].

These advantages documented in higher education have led to the expansion of BID instruction into K-12 engineering education. As Nagel et al. [6] reported, students completing a BID curriculum felt they learned more about engineering by connecting engineering to their pre-existing biology content [9]. Bio-inspired design within the K-12 setting is also associated with positive student perceptions of the importance of nature [6,8]. While recent efforts have brought BID into the K-12 space, it has not been fully incorporated into K-12 engineering education [6,9,10,11,12,13]. For students to reap the benefits of BID integration, it is imperative that teachers feel knowledgeable and efficacious about teaching BID in engineering classrooms. It is, therefore, critical to develop and provide teachers with rich and meaningful professional learning experiences to strengthen their understanding of BID integration in engineering.

This study is part of a larger project centered in the K-12 (high school) setting, which created socially relevant, accessible, and highly contextualized high school engineering curricula focusing on BID [10,11,12,13]. The professional learning experience was designed and implemented for high school engineering teachers during the summers of 2020, 2021, and 2022 [10,11,12]. In this paper, we investigated the environment designed to foster these teachers’ understanding of BID integration in engineering and to determine to what extent the evolving environment fostered engineering teachers’ conceptual knowledge of BID across the three years of experience. This work builds on a study that investigated high school engineering teachers’ experiences across two years [11]. In the context of the three-year case study, this study expands on prior analysis and offers new insights about the learning environment needed to support teacher professional development in the context of BID. This research also demonstrates a nascent method in education research that combines conjecture mapping with design-based research and a method for retrospective analysis that may be particularly useful in evolving, convergent fields such as BID.

## 2. Background

### 2.1. Professional Learning Experiences

Research demonstrates many advantages of professional learning experiences [14]. Professional learning is defined as ‘a product of both externally provided and job-embedded activities that increase teachers’ knowledge and change their instructional practice in ways that support student learning’ [15] (p. 3). Professional learning environments that are inquiry- or learner-centered support teachers as they collaboratively develop the professional knowledge they need to use in their context [16]. In recognition of their effectiveness, professional learning for teachers has increased to facilitate educational improvement worldwide [16].

Experiential learning is a particularly effective component of professional learning [14,17]. Rooted in the works of Dewey, Piaget, and Vygotsky, it involves constructing knowledge and meaning from real-life experiences [18,19,20]. This process of change places teachers in the role of a learner before they teach their students, encouraging professional and personal growth [19,20] by reflecting on their instructional styles and working to meet learner needs [19,20]. Each learner’s experience is unique, enabling them to ground their past experiences as a foundation to engage with the new [21]. Further, by engaging in self-evaluation and critical thinking, teachers can better meet their students’ needs [21,22]. Experiential learning, when utilized in professional learning, expands teachers’ pedagogical practices and aids in developing their conceptual understanding, which can impact their students’ learning [19,23,24].

### 2.2. Purpose

The project associated with this research developed, implemented, and supported a curriculum that incorporates BID into the engineering design process within a high school setting. The curriculum spans two 7-week units to be implemented over two years in the first two high school engineering courses of a 3-course engineering sequence. Given the span and depth of developed content and the diverse and traditionally departmentalized nature of the underlying content, multiweek professional learning was designed to facilitate teachers’ understanding of BID pedagogy and unique methods, build biological and engineering content knowledge and self-efficacy, demonstrate integration with traditional engineering processes, and provide hands-on experience with lessons of curriculum implementation, including planning and classroom preparation. These professional learning experiences for teachers were hosted each summer for three consecutive years (i.e., Summer 2020, 2021, and 2022).

This study examines the changing professional learning environment over three years and its impact on teachers’ conceptual understanding of BID integration in engineering using a combined method of DBR and conjecture mapping. Conceptual understanding is teachers’ ability to construct meaning, to interpret, to apply, and to explain the learned concepts [25]. Conceptual understanding, as defined in the science education literature, involves understanding ‘of the principles that govern a domain and of the interrelations between units of knowledge in a domain’ [26] (pp. 346–347). In this paper, we assess whether the key elements incorporated in the iterative professional learning implementations allowed engineering teachers to better understand BID integration in engineering design than the prior professional learning instances. We aim to address the following research question: How does the changing professional learning environment impact teachers’ conceptual understanding of BID integration in engineering across three years of summer professional learning?

### 2.3. Methodological Background

The approach to analysis is grounded in design-based research (DBR) with conjecture mapping [27]. DBR is the study of learning through the design, implementation, and subsequent study of innovative learning experiences and instructional strategies [28,29]. DBR allowed researchers to examine and refine the learning environment over multiple years of implementation [11,30,31]. Iterative cycles of design, enactment, analysis, and redesign are central to DBR methodologies [29,32]. This iterative cycle supported the development and refined design conjectures [27]. Therefore, we will leverage iterative conjecture mapping to frame and understand the three iterations of the professional learning environment for the teachers. Conjecture mapping provides a method of investigating causal processes of how the professional learning design supported the desired outcomes through an explicit focus on interactions with design elements [27]. In this way, conjecture mapping can be used to connect teacher outcomes through iterative design cycles.

#### 2.3.1. Design-Based Research (DBR)

DBR is a practical methodology for producing new theories and practices potentially impacting learning and teaching in naturalistic settings [11,28,33]. In DBR, there is an intentionality in the research process to refine theory and practice [33]. DBR interventions embody theoretical claims about teaching and learning and aim to understand the relationship among theory, designed artifacts, and practice [32,33,34]. DBR recognizes that designing learning environments is often a theoretical activity that embodies hypotheses about how learning occurs within the produced context and how that learning might best be supported [28,32]. Therefore, DBR is essential because it recognizes that neither theory nor interventions alone are sufficient [11,26,30]. The fundamental guiding principle of DBR is the study of ‘learning as situated within a learning ecology’ [34] (p. 3). In this project, the learning ecology is the professional learning environment, encompassing activities, tools, resources, and the participatory and pedagogical processes the engineering teachers engaged in during their three-year professional learning experience [10,11,34].

#### 2.3.2. Conjecture Mapping

**Figure 1:**
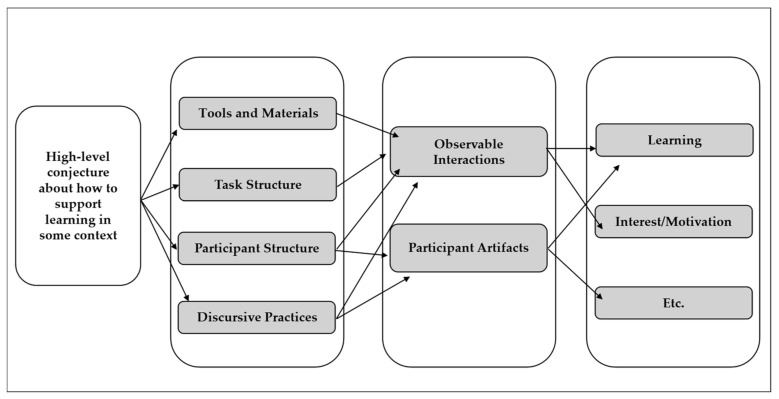
Generalized conjecture map for educational design research [27].

Conjecture mapping is a tool designed to support researchers in systematically developing an educational innovation that addresses specific teaching and learning goals [27]. At its core, conjecture mapping involves four main elements. The first element involves a high-level conjecture, which is an abstract idea about the learning principles selected in the design of the learning environment to support the desired outcomes [11,27,34]. This high-level conjecture is intended to be met in a learning environment via the second element, referred to as specific embodiments, as illustrated in Figure 1 [11]. The embodiments include tools and materials, task structures, participant structures, and discursive practices [27] and are often referred to as the main characteristics of the design [27,34]. The embodiments generate mediating processes (third element) that involve observable interactions generated through learning activities shared between participants and the environment. The mediating processes are the ‘mechanisms of the study that connect the conjectures to the desired outcomes’ [34] (p. 145). These mediating processes produce outcomes (fourth element) [27]. A learning environment is deemed successful if the outcomes generated support the high-level conjecture initially set for the project. When determining if and to what extent outcomes are met, the connections between design, conjectures, and design components are considered [35,36].

#### 2.3.3. Integration of DBR and Conjecture Mapping

**Figure 2:**
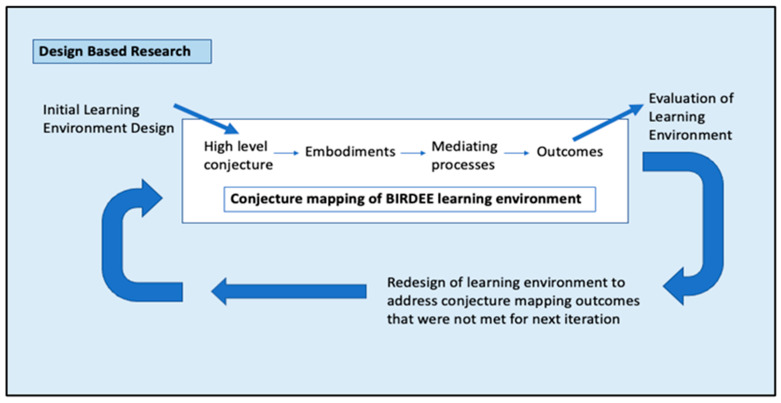
An evaluation cycle using design-based research and conjecture mapping.

Conjecture mapping can be supported by and can support DBR since conjectures support the iterative testing process used to examine and revise the learning environment (Figure 2) as it is designed and subsequently implemented [32,35]. DBR research emphasizes authentic, applicable contexts for research [32,34] that are often changing, especially in the ‘context of engineering problems’ [34] (p. 144). A conjecture map makes the design of the intervention in DBR more explicit by forming pathways that connect the design of a learning environment to the desired outcomes. Moreover, conjecture maps aid in visually illustrating and explaining the changes in the intervention that can potentially refine theory and practice in DBR [27].

## 3. Materials and Methods

### 3.1. Participants and Settings

The participants were seven high school engineering teachers, including five males and two females. Of the seven participants, three identified as White, three as Black and African American, and one as Asian. The highest educational level for five of the teachers was a master’s degree, while two obtained a doctorate in engineering. Teachers ranged from first-year novices to ten or more years of experience (See Table 1). All teachers were certified to teach 6–12 engineering [10,11,12,13]. Of the participants, one teacher participated in all three years of professional learning, one for two consecutive years, and the rest participated in one of the three years.

The seven participating teachers represented five high schools from two school districts in a large southeastern metropolitan area. Schools’ demographics varied; in three high schools, the majority of students were Black or African American, representing between 89% and 96% of the student population. In the fourth school, the majority of students were White, representing 57% of the population, while the remaining students were Hispanic (20%), African American/Black (16%), and Asian (4%). The fifth school was a STEM-focused school where the student community was more diverse: 37% were White, and the remaining students were Asian (37%), Black (13%), Hispanic (8%), and Multiracial (5%). All the schools were public high schools [10,11,12,13]. The teachers were recruited from school districts that had agreed to participate in the project. Participants included all teachers in those districts who accepted our invitation and were willing to implement the curriculum in their classrooms [10,11,12,13].

### 3.2. Context: Professional Learning

The professional learning experiences were initially conceptualized to be face-to-face at the host university as part of a six-week summer internship at university research laboratories focused on biology and biologically inspired design [10,11]. As the COVID-19 pandemic resulted in university closures in the summer of 2020, the project team transitioned from face-to-face instruction to an online setting. An online meeting platform, Blue Jeans, was used for meetings, and all artifacts/assignments and readings were shared with teachers via the Canvas online learning platform. Though online, the goal remained the same: to connect engineering teachers to the natural world through immersive experiential learning activities to improve teachers’ knowledge of bio-inspired design integration in engineering. A synchronous, quasi-facilitated online course allowed participants to interact in real-time for over six weeks [10,11].

Professional learning was designed to engage teachers in experiential learning, transforming experiences into active and effective learning through Kolb’s [20] 4-stage cycle to promote understanding (see Table 2). Experiential learning enabled teachers to engage as learners in direct experiences and reflections to foster their understanding of BID, the engineering design process, and pedagogy.

Teachers completed multiple activities ranging from group discussion, visits to the zoo and botanical gardens, structure, function, mechanism (SFM) analysis, found object discussions, and reflections on curriculum activities. Core activities that were implemented across the three years are presented in Table 3.

In year one (summer of 2020), teachers attended the online, synchronous professional learning for two hours two days a week (Tuesdays and Thursdays), in addition to offline, asynchronous assignments. These assignments included virtual tours of the zoos, engagement in group collaboration, discussion, and reflection. Structure, function, and mechanism were introduced to aid in evaluating biological solutions, and a final design challenge culminated in teachers applying what participants learned to produce a biologically inspired vaccine transport device [10,11,37].

In year two (summer of 2021), teachers attended professional learning activities each day either synchronously online for approximately two hours or in-person for up to six hours over four weeks, with a two-week hiatus in the middle due to teacher availability. The hybrid’s duration was shorter than the previous year in terms of weeks. Still, the daily and longer in-person meetings allowed a similar amount of content to be delivered, although spaced differently. Teachers still had an opportunity to discuss, collaborate with their peers, and reflect. The activities for the second professional learning included replicating potentially challenging curriculum activities, increased focus on SFM, found object analysis, and in-person field trips to the botanical garden, zoo, and research laboratories (see Table 2), but the design activity was removed in favor of the curriculum activities and SFM focus. Teachers participated in activities from the student perspective, completing written reflective questions and presenting their work within the online sessions.

The third professional learning (summer of 2022) was also hybrid and divided into virtual and face-to-face learning. Teachers attended the professional learning activities face-to-face for eight hours for two of the six weeks, with a one-week hiatus in between. The virtual and face-to-face learning rotated each week, with the first week being virtual and the final week face-to-face. In year three, the professional learning included more specific curriculum activities and SFM analyses to help teachers understand and evaluate biological solutions. Teachers and the project team again visited the zoo, botanical gardens, and several bio-inspired research laboratories, and similar to year one, they engaged in a final design challenge that culminated in applying what they learned to produce a biologically inspired vaccine transport device. The environment continued to engage participants in the activities from a student’s perspective and then reflect from a teacher’s perspective.

### 3.3. Data

The data sources for this three-year study included teacher artifacts, researcher field notes, and semi-structured teacher interviews [10,11]. Additionally, all online and classroom-based professional learning sessions were recorded across the three years. The weekly topics of the recorded learning activities are observed in Table 4: professional learning weekly schedule across the three years.

Professional Learning Artifacts. Professional learning was designed to engage teachers in experiential learning, and therefore, teachers completed multiple activities (See Table 3). The professional learning artifacts included an analysis of a found object in nature, for which teachers annotated sketches of three found objects, identified a well-articulated function of interest, and provided functional and mechanistic descriptions of one function of interest. Teachers also completed written reflections on teaching practices and professional learning activities at the end of each session. In addition, in years one and three, teachers completed a design challenge activity in which they worked to design a vaccine transport device. The engineering design process was documented in the engineering design process logs. Lastly, the project research team took field notes during the professional learning to document the facilitators’ and participants’ conversations and interactions [10,11,12].

Semi-structured teacher interviews. Semi-structured teacher interviews were conducted at the end of the professional learning across all three years [10,11,12]. The semi-structured interviews were designed to elicit teachers’ views regarding professional learning, specifically the structure, the learning activities, BID integration in engineering, the engineering design process, and curriculum implementation in their respective classrooms. The project research team conducted the interviews online. The interviews took approximately 45 min to complete. All the interviews were recorded and transcribed [10,11,12].

### 3.4. Analysis: Combined DBR and Conjecture Mapping Process

**Figure 3:**
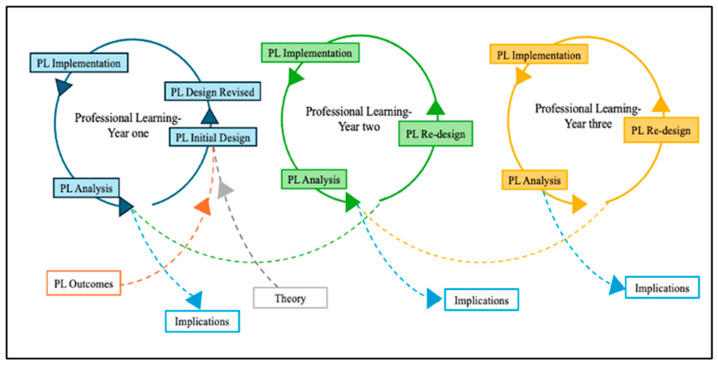
Embedded design cycles of the professional learning across the three years.

To address the research question, the analysis followed the various stages described below and depicted in Figure 3. An inductive qualitative analysis process was employed to examine, categorize, and code the data [38] to identify the embodiments (i.e., tool and structure, task and participant structures, and discursive practices), the mediating process (i.e., observable interactions and participant artifacts), and how these connected to achieve the intended outcomes. The data were analyzed to determine how tools and materials, task and participant structures, discursive practices, and mediating processes applied across professional learning supported teachers’ understanding of BID integration in engineering teaching. This was then utilized to develop conjecture maps based on our high-level conjecture, a commonly used approach in DBR [10,27,32,39]. The conjecture map was reviewed, revised, and discussed several times until the researchers reached an agreement on all of the critical aspects of the professional learning’s design, embodiments, and mediating process [27]. The iterative DBR design cycles were then used as a lens for interpreting and revising the conjecture maps developed for the professional learning environment designed for teachers (Figure 3).

The professional learning experiences were analyzed and presented as an iterative sequence of evolving conjecture maps across three years to illustrate the changing professional learning environment and its impact on teachers’ conceptual understanding of BID integration in engineering. The conjecture map highlights the high-level conjecture and the desired project outcomes of the professional learning to illustrate if the embodiments and the meditated processes each year facilitated teachers’ integration of BID into their pre-existing teaching engineering schema due to their engagement in experiential learning and interactive discussions [27]. Following the conjecture map, an explanation is provided to highlight the factors that may have contributed to teachers’ understanding of BID integration or lack thereof. The following abbreviated identifier is used when quoting from the data: ‘T#’ for teacher identification.

## 4. Results

**Figure 4:**
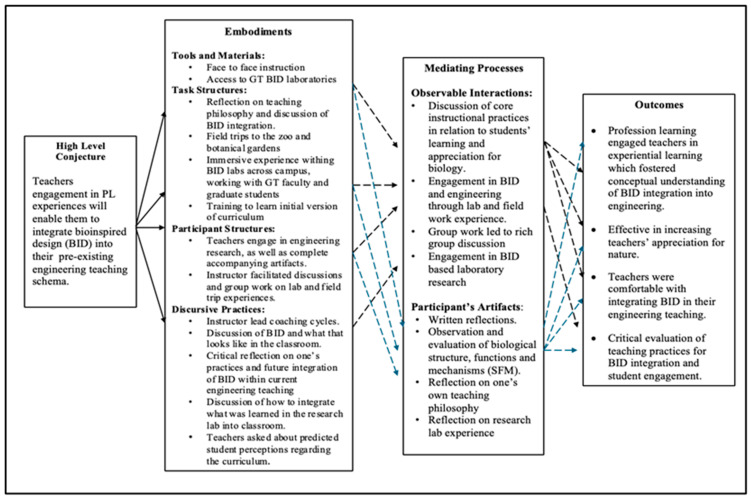
Original conjecture map of the professional learning environment.

The professional learning was intended to connect teachers to the biological world through an immersive experiential learning environment, which included visits to the botanical gardens and zoo, to improve teachers’ conceptual understanding of bio-inspired design integration in engineering. While the original professional learning was planned to be face-to-face (see Figure 4), it was quickly shifted to a virtual format due to COVID-19 restrictions. Based on the original design, project researchers made an initial conjecture map before data analysis, which was then used to test design ideas, and data were used to refine the conjecture map. Figure 4 depicts the initial conjecture map of hypothesized connections between professional learning design and outcomes.

### 4.1. Professional Learning Year One

**Figure 5:**
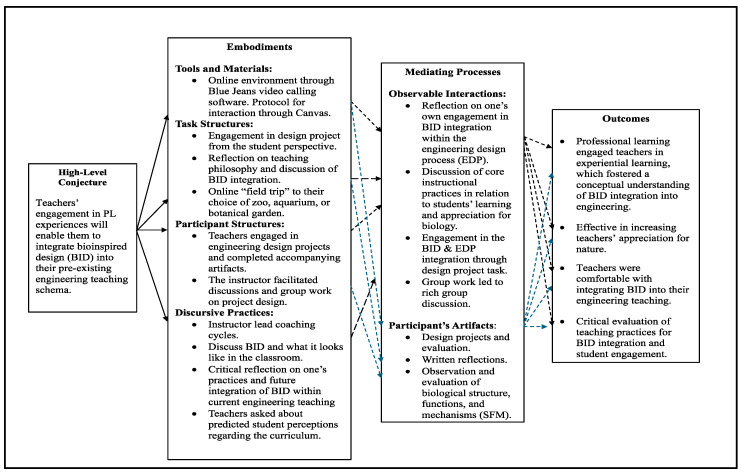
The 2020 professional learning environment conjecture map [11].

The first year of professional learning in 2020 was shifted to an entirely virtual learning platform (Canvas), utilizing Blue Jeans software for cloud-based video conferencing (see Figure 5). In year one, the overall goal was for teachers to be able to integrate BID within their pre-existing schema for engineering teaching as a result of their engagement in professional learning. Consequently, teachers engaged in a research design project intended to incorporate BID within the design of a vaccine transport device. Teachers and the instructional team discussed various topics, such as teachers’ pedagogical approaches and teaching philosophy, BID integration within the engineering design process, and teachers’ thoughts on probable student perceptions of the presented material. Finally, teachers participated in group discussions, working together to ideate and develop their design solutions, including structure-function–mechanism (SFM) analyses of biological objects.

The findings revealed that, overall, professional learning fostered teachers’ conceptual understanding of BID integration within the engineering design process. Additionally, teachers’ appreciation for nature and comfort with BID increased, as one teacher indicated, ‘The investigation portion was fun in that it caused me to think about what I was actually looking for and some of the different functions of the insect, plants, etc. that are around me’ (T#1). Another teacher indicated, ‘How problems can be solved by implementing these concepts...the possibilities are endless’ (T#2). However, it should be noted that although teachers felt comfortable with the ‘big picture’ conceptual understanding of BID integration within the engineering design process, they expressed hesitancy regarding the specific tools for biological object analysis, such as SFM breakdown. This hesitancy may be attributed to their biological content knowledge and their limited exposure to the SFM analysis.

Furthermore, teachers reflected on their teaching practices and what they believed would be best for student engagement and BID integration. Many teachers indicated that information needed to be simplified for students to grasp these concepts. For instance, a teacher stated, ‘I always reinforce or taught [sic] that once you break a problem down into increments, it is no longer hard’ (T#2). Similarly, another claimed that the best way to engage students and help them learn these concepts is through stories and examples. The examples provided to the students ‘help them grasp the magnitude of inspiration that [they] can get from nature’ (T#1). While the first year of professional learning was largely successful, there was still room for improvement, particularly regarding teachers’ ability to conduct breakdowns of biological objects and their structure, function, and mechanism. Also, the completely virtual environment prevented the teachers from engaging in an immersive experience necessary for BID understanding. Thus, we aimed to address these areas in year two through the DBR approach, as described below.

### 4.2. Professional Learning Year Two

**Figure 6:**
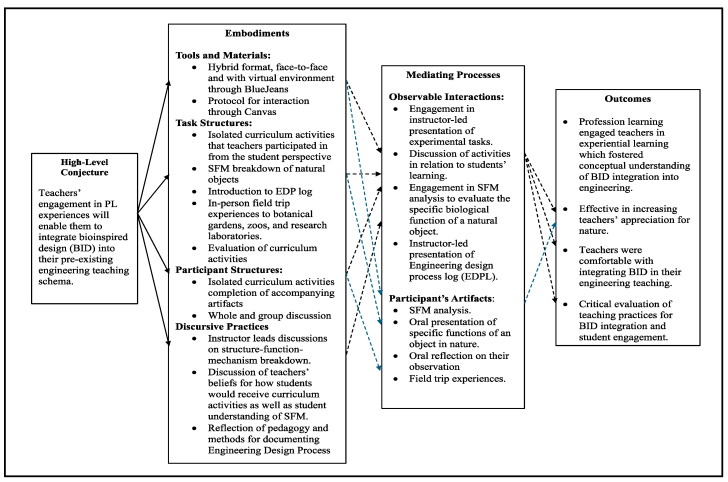
The 2021 professional learning environment conjecture map [11].

For the second year, changes were made to address these areas of improvement, especially focusing on teachers’ ability to analyze biological objects’ structure, function, and mechanism. The changes encompassed shifting the virtual professional learning to a hybrid format, instructor-led discussions on structure–function–mechanism breakdown, and engagement in curriculum-based activities. Also, because the instructional team was simultaneously developing the curriculum, teacher activities focused more on extensive coaching for structure–function–mechanism breakdown while testing specific activities intended for the curriculum (Figure 6).

The findings revealed that in year two, three out of the four intended outcomes were met. The hybrid format, compared to the entire virtual structure from the first year, enabled teachers to engage with in-person activities and field trip experiences while having some flexible virtual workdays. The mixed format was more enjoyable for the teachers. It fostered enthusiasm for professional learning, as one of the returning teachers indicated that ‘compared to the first year, [this professional learning] was much more engaging due to the in-person aspect’ (T#3). Similarly, another suggested, ‘Well, it was more engaging, so the fact that you have the human interaction definitely made it more rewarding and the learning experiences easier’ (T#4).

Additionally, teachers were more comfortable with the breakdown of biological entities as one teacher claimed, ‘I really think that this summer’s [professional learning], I got it! Moreover, I feel comfortable implementing it. Last summer, I only knew it as a term [referring to SFM]. I really feel that I now know how to look at the biology portion of it. Last year, I would have thought that we needed an entire science class’(T#2). However, due to the shift of focus away from the conceptual idea of integrating BID within the engineering design process, many teachers were unable to articulate how BID was incorporated into the engineering design process. For instance, one teacher claimed about a curriculum-specific activity [elephant gripper activity] that ‘our focus was on modifying the gripper if we had been working on the actual build, then that is where we should use inspiration from elephant trunk’ (T#3), suggesting that teachers did not see the application of BID within the given activity or the iterative and fluid nature of engineering design. Overall, because teachers were unfamiliar with implementing BID within the engineering design process in a regular design project, they had difficulty understanding how the integration might work in their classrooms. Additionally, teachers did not discuss their teaching practices to the same extent as the previous year due to the focus on the more technical aspects of BID.

### 4.3. Professional Learning Year Three

In the third and final year, embodiments were again altered to better meet the intended outcomes. These alterations resulted from the lessons learned from the previous year (2021), such as the hybrid professional learning format, teachers’ engagement in a culminating design challenge, and interactive discussions among teacher participants.

**Figure 7:**
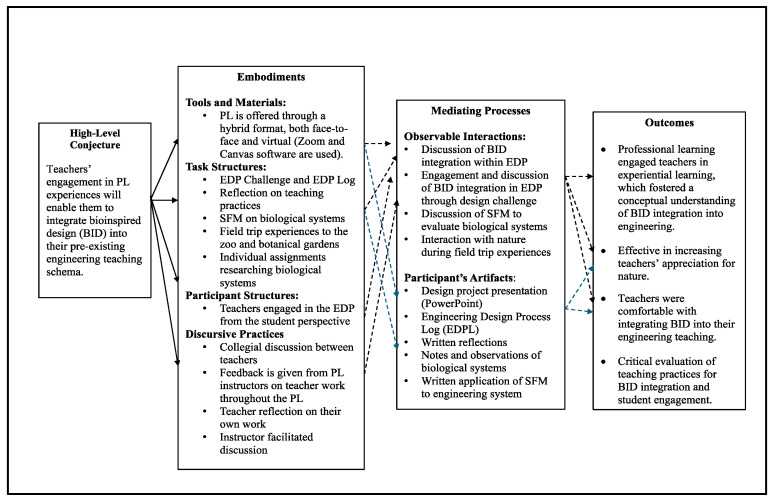
The 2022 professional learning environment conjecture map.

In the final year, effective prior year’s professional learning components were integrated with new components to address the intended outcomes better. First, the format for delivery remained the same, as a hybrid platform was found effective in the previous year. The task structures differed significantly from the previous year, as teachers worked on a culminating engineering design project (returning to the same project from the first professional learning) and completed the engineering design process log. The decision to engage teachers again in a culminating engineering design project derived from their lack of conceptual understanding of the BID integration in engineering due to more emphasis on SFM and engineering design process log and specific heavily structured curriculum activities in the previous year. For discursive practices, teachers engaged in discussions with their colleagues and guided discussions held by the instructional team. Teachers also participated in in-person field trip experiences to a local zoo and botanical garden (Figure 7).

Due to the changes in embodiments, the mediating processes were also altered. This third year resulted in discussions around BID integration within the engineering design process, what classroom integration should look like with perspective to the teachers’ design challenge, and documentation for the engineering design process log. While the discussions were rich in regard to content and practices, they lacked critical evaluation of student engagement in BID due to many teachers attending professional learning for the first time. Teachers also interacted with nature through field trips and the found object exercise. The resulting artifacts included the engineering design presentation and the engineering design process log used to document their progress, as well as the teachers’ written reflections, notes, and SFM analyses of biological systems.

Outcomes for this final professional learning were better met than the year prior. Teachers were engaged with the entire engineering design process throughout the professional learning and successfully integrated BID components into their engineering design projects. As observed during the professional learning discussions, teachers highlighted potential design solutions incorporating BID components, such as the alligator snapping turtle, plant leaf structure, shark scales used to reduce disease transmission, and BID integration within hovercraft design. The final design solutions that the teachers created in response to the vaccine transport prompt also incorporated BID aspects in their design solutions and were more detailed than the previous year (year one).

Moreover, the teachers’ appreciation for nature was also heightened through their field trip experiences. They began to view the natural world around them as a potential source of inspiration for human problem-solving. Teachers expanded their thinking and knowledge about nature as they learned about BID integration in engineering design. This was emphasized during the professional learning discussions, where one teacher when speaking about BID, stated, ‘I had not thought about the plants in that perspective’ (T#4). Additionally, in the final interviews, that same teacher described BID ‘as another way of thinking’ (T#4). When probed further regarding the role of SFM in BID and engineering. The teacher claimed, ‘SFM is inside the BID… you must have a biological structure… and the breakdown of BID is SFM’ (T#4). Another teacher described in his interview that he thought the curriculum would help students see nature differently, speaking to the project’s potential impact (T#5). In year three, the formal (i.e., design project) and informal (i.e., field trips) experiences enabled teachers to immerse themselves in real-world interactive experiences that supported teachers’ conceptual understanding of BID integration in engineering.

## 5. Discussion

The findings of this study highlight the unique needs of high school engineering teachers when integrating BID in their classrooms. Teachers’ content knowledge plays an essential role in curriculum implementation [40,41], all the more so in convergent fields such as BID, where teachers require the traditionally departmentalized knowledge of both engineering and biology to integrate BID effectively in their classrooms [40,41]. The findings of this research extend the existing literature on BID-integrated teacher learning experiences by connecting the design of a learning environment with teacher outcomes through specific embodiments and mediating processes to support the unique needs of teachers in learning about BID and feeling confident with teaching BID [10,11,18,19,42]. This study documents the best practices for integrating BID by engaging teachers in experiential learning that includes a combination of formal and informal experiences within an engineering-focused professional learning context, summarized in three key findings. A fourth finding suggests a method for both designing and studying such experiences.

The first finding highlights that experiential learning, which enables teachers to experience through a student’s lens, was critical for teachers to develop an understanding of BID integration within the engineering design process. The professional learning encompassed several activities and experiences that engaged teachers in hands-on and interactive learning, such as the zoo field trips and found object activity. Specifically, the culmination design project (years 1 and 3) and the found object activity (years 1, 2, and 3) both provided teachers with meaningful learning from the perspective of student learners, which aided in developing teachers’ conceptual understanding of BID integration in engineering. Teachers must achieve conceptual understanding before they can thoughtfully instruct their students, reinforcing the need for high-quality professional learning experiences [18,43]. These activities also aided teachers in understanding teaching practices and facilitated more robust predictions for student needs. Experiential learning activities that encourage active learning provide individuals with ‘an opportunity to experience concepts first-hand and, as such, give students a richer, more meaningful understanding of course concepts and of how they operate in the real world’ [44] (p. 594). Therefore, professional learning should be driven through active learning experiences that allow participants to build their understanding [21,43,45].

The second finding indicated that the hybrid professional learning format combining remote and in-person learning contributed to teachers’ understanding and engagement in BID. Initially, COVID-19 limited teachers’ engagement in in-person learning, particularly in year one. However, as professional learning shifted to a hybrid format (years 2 and 3), employing both online (synchronous and asynchronous) and in-person activities to facilitate teachers’ learning, it better supported peer interaction and expert-led exploration of biological objects, making learning more meaningful for the teachers. The hybrid format also allowed teachers to absorb the content provided at their own pace, engage in knowledge-sharing activities, and reflect on their teaching practices and content. The hybrid format further supported the human connection that is necessary to foster active learning, rich discourse, and collaboration among colleagues and experts. A hybrid professional learning environment must foster rich interaction [45,46] and engage learners in discussions and experiences that support cooperative, contextualized engagement to develop a deeper understanding of the content.

The third finding suggests that both formal and informal learning were beneficial in developing teachers’ understanding of BID in engineering. The informal learning experiences (e.g., zoo, botanical gardens, and university lab visits) fostered enthusiasm for BID integration and offered impactful opportunities for learning and ideating with experts. These immersive experiences ground BID understanding to tangible, real-world examples that serve as reference points for novice learners. The learning during such experiences was transferred back into the formal learning space through group discussions, reflection, and the incorporation of ideas into engineering design challenges. This coupling of informal and formal experiences advanced teachers’ understanding of the integration of real-world biology within engineering. Melber et al. [47] suggest that the inclusion of informal experiences in teachers’ professional learning ‘provides the virtually untapped potential to engage teachers in professional enhancement that integrates professionalism, content, and pedagogy’ (p. 105). A combination of formal and informal learning experiences has the potential to significantly enhance teachers’ development and conceptual understanding of the concepts.

The fourth finding is about the method of design and study. The use of conjecture mapping coupled with DBR provides visual design conjectures to demonstrate how the DBR paradigm influences development over time [27,34,48,49]. This research provides one example of how conjecture mapping and DBR can be used to visualize continual improvements to a professional learning environment and how this combination of tools can proactively assess the quality of a learning environment within many educational settings and inform decision-making to meet the intended outcomes iteratively [27,30,32,34,35]. 

## 6. Conclusions

This research is centered within a K-12 setting and is focused on teacher experience resulting from a BID professional learning environment across three years. Specifically, we investigated how the learning environment led to integrating BID within engineering teachers’ pre-existing schema for pre-college engineering education. This study highlights the importance of experiential professional learning experiences for teachers that combine informal and formal learning experiences to aid in developing their conceptual understanding of BID in engineering. Moreover, the use of conjecture mapping coupled with design-based research methodological grounding demonstrates critical causal linkages between professional learning adaptations and outcomes in post hoc analysis, for example, identifying the critical elements that contributed to developing teachers’ conceptual understanding of BID integration in engineering, such as immersive formal and informal experiences coupled with a design project. Our results also support DBR and conjecture mapping for proactive use to understand, align, and refine the professional learning environment to meet objectives, especially in dynamic environments such as those encountered during COVID. Finally, this study supports the inclusion of immersive learning experiences (e.g., research lab visits and nature walks) to advance teachers’ understanding of bio-inspired design integration in engineering in the context of professional learning.

## 7. Limitations

This study’s limitations include sudden alterations made to professional learning due to the COVID-19 pandemic, driving a lack of curriculum implementation within K-12 classrooms. Additionally, teacher retention and burnout impacted our study as teachers left or switched schools across the three years of professional learning and implementation.

## Figures and Tables

**Table 1:** Participating teachers’ demographic characteristics.

Category	Subgroup	Number of Teachers
Gender	Male	5
	Female	2
Race	White	3
	Black/African American	3
	Asian	1
	Other	0
Highest Education Level	Bachelor’s	0
	Master’s	5
	Doctorate	2
Years of Experiences	0–2 years	2
	3–5 years	2
	6–10 years	0
	>10 years	3

**Table 2:** Professional learning activities mapped to Kolb’s cycle.

Experiential Learning Cycle	Description	Activities
Concrete Learning	The ‘doing’ phase, where learners immerse themselves in an activity without prejudgment or preconceived ideas.	Field trips to zoo and botanical gardens; structure, function, mechanism (SFM) analysis.
Reflective Observation	The ‘observing’ phase, where one’s actions and the results allow space for introspection and evaluation.	Pedagogical and content discussion; completion of content reflections.
Abstract Conceptualization	The ‘thinking’ phase, where learners reflect about and process the reflection into a new idea or concept.	Planning for curriculum implementation, engagement in engineering design process (EDP) to ideate solution.
Active Experimentation	The ‘planning’ phase, where learner applies the concepts learned in the previous stage to new situations.	Application of BID into their design solution.

**Table 3:** Central activities within the professional learning experience.

Activity	Description
Found Object	Participants are asked to investigate a common biological object found close to the individual’s home or frequented environment. This analysis investigates an everyday biological object that the teacher had not considered from an engineering perspective, with attention to *function* and the *structures* that work together to produce said function (*mechanism*). This activity introduces participants to the ubiquity of engineering principles in nature and provides experiential grounding for future pedagogical elements.
Structure– Function– Mechanism (SFM) Analysis	Participants use the SFM modeling framework to document and describe functions of a complex systems. For each function examined, the analysis calls for identification of the structures that perform that function, as well providing a causal account of how the structures give rise to the function (mechanism).
Field Trip Experiences	Field trips to a zoo, botanical garden, and research laboratories allowed participants to observe biology in-person and engage with experts at each location. Participants were encouraged to ask questions and participate in discussions related to biological organisms. The experience was designed to increase biological content knowledge, provide knowledge of biological organisms for use in design activities, and create grounded experiences to facilitate future interactions with students.
Vaccine transport engineering design challenge	Participants were asked to design an ideal vaccine transport device engaging using the biologically inspired design process as students, including completion of associated curricular materials.

**Table 4:** Professional learning weekly schedule across the three years.

Weeks	Year One	Year Two	Year Three
Week 1	Intro to Bio Inspired Design—Found Object Activity	Exploration of nature: curriculum overview, reflection on teacher practices, and experimental activities	Discussion of BID integration with current projects; found object exercise and teacher reflection
Week 2	Design Challenge Part A: Problem Definition and Understanding	Unit 1 review continued, completion of curriculum activities, discussion of women in engineering, structure (SFM) analysis	Trip to zoo and botanical gardens; BID integration (SFM); design challenge introduction
Week 3	Design Challenge Part B: Ideation, Biological Analogies to Evaluate Biological Solutions	BREAK	BREAK
Week 4	Independent Work: Nature Walk and Test Planning: Found Object Investigation in Relation to SFM	BREAK	EDPL review; working on design challenge; design challenge check-in
Week 5	Design Challenge Part C: Prototype Planning, Fabrication and Curriculum Review; Prototype Development	SFM analysis and Reflection: zoo visit, exploration of a natural object, and SFM overview	Unit 1 curriculum activities (lotus effect, morpho matrix); Introduction to unit 2 activities; design challenge solutions presentations; lab tours
Week 6	Design Challenge Part: Testing/evaluation of Prototypes and Final Presentation	Lab visits, reflection on found object, pedagogical training, engineering log (EDPL) completion	Reflection on PD and planning for classroom implementation

## Data Availability

All relevant data are included within the paper.
